# Association and dose-response relationship between exposure to alcohol advertising media and current drinking: a nationwide cross-sectional study of Japanese adolescents

**DOI:** 10.1265/ehpm.23-00127

**Published:** 2023-09-28

**Authors:** Keita Yoshida, Hideyuki Kanda, Takashi Hisamatsu, Yuki Kuwabara, Aya Kinjo, Hisashi Yoshimoto, Teruna Ito, Hideaki Kasuga, Ruriko Minobe, Hitoshi Maesato, Maki Jike, Yuuki Matsumoto, Yuichiro Otsuka, Osamu Itani, Yoshitaka Kaneita, Susumu Higuchi, Yoneatsu Osaki

**Affiliations:** 1 Department of Public Health, Okayama University Graduate School of Medicine, Dentistry, and Pharmaceutical Sciences, 2-5-1 Shikata-cho, Kita-ku, Okayama, Okayama 700-8558, Japan; 2 Division of Environmental and Preventive Medicine, Department of Social Medicine, Faculty of Medicine, Tottori University, 86 Nishi-machi, Yonago, Tottori 683-8503, Japan; 3 Department of Family Medicine, General Practice and Community Health, Institute of Medicine, University of Tsukuba, 1-1-1 Tennodai, Tsukuba, Ibaraki 305-8575, Japan; 4 Department of Food and Nutrition, Koriyama Women’s University, 3-25-2 Kaisei, Koriyama, Fukushima 963-8503, Japan; 5 Department of Hygiene and Preventive Medicine, Fukushima Medical University, 1 Hikarigaoka, Fukushima, Fukushima 960-1295, Japan; 6 National Institute of Alcoholism, Kurihama National Hospital, 5-3-1 Nobi, Yokosuka, Kanagawa 239-0841, Japan; 7 Department of Food Science and Nutrition, Faculty of Life and Environmental Science, Showa Women’s University, 1-7-57 Taishido, Setagaya, Tokyo 154-8533, Japan; 8 Division of Public Health, Department of Social Medicine, Nihon University School of Medicine, 30-1 Oyaguchi-kamimachi, Itabashi, Tokyo 173-8610, Japan

**Keywords:** Underage drinking, Alcohol, Adolescents, Advertisement

## Abstract

**Background:**

Underage drinking is a public health concern. However, few studies have examined the association between alcoholic beverage advertising and underage drinking, particularly in countries with low underage drinking rates, such as Japan. Therefore, we aimed to investigate the relationship between exposure to advertising in various media and alcohol drinking among Japanese adolescents.

**Methods:**

We conducted a cross-sectional study involving 15,683 adolescents (51% girls) using data from a nationwide lifestyle survey in 2021 among junior and senior high schools across Japan. Media types were websites, stores, and public transportation. We defined current drinking as alcohol consumption of ≥1 day in the 30 days preceding the survey. Multivariable logistic regression was used to examine the association between exposure to alcohol advertisements and current drinking, adjusting for sex, grades, school area, lifestyle (bedtime and having fun at school), and addictive behaviors (smoking status and parents’ alcohol consumption).

**Results:**

The prevalence of current drinking was 2.2% (2.3% of boys and 2.0% of girls). Students who were exposed to any alcohol advertising media had higher odds of current drinking compared with those who were not (odds ratio, 1.48; 95% confidence interval [CI], 1.18–1.87). Students who were exposed to web, in-store, and public transportation advertisements had odds ratios of 1.44 (95% CI, 1.14–1.81), 1.62 (1.28–2.05), and 1.45 (1.06–1.98) of current drinking, respectively, compared with those who were not. The association of exposure to alcohol advertising media with the prevalence of current drinking was similar among boys and girls (all *p* for sex interaction >0.1), except for that of exposure to web advertisements; its association with current drinking was more pronounced in girls (*p* for sex interaction = 0.046). Exposure to a larger cumulative number of different alcohol advertising media was independently associated with a higher prevalence of current drinking among all students, boys, and girls (*p*-values for trend <0.001, 0.031, and <0.001, respectively; *p* for sex interaction = 0.085).

**Conclusions:**

We found an association with a dose-response relationship between exposure to alcohol advertisements and current drinking among adolescents in junior and senior high schools across Japan. Our findings highlight the need for further advertising regulations to prevent underage drinking.

**Supplementary information:**

The online version contains supplementary material available at https://doi.org/10.1265/ehpm.23-00127.

## 1. Introduction

Underage drinking is a public health issue because of its negative effects (e.g., violence, mental disorders, and risk of alcohol dependence) [1–4]. The global strategy to reduce the harmful use of alcohol was adopted at the World Health Organization General Assembly in 2010. Adolescents were mentioned as a population at particularly high risk for the harmful use of alcohol. The prevalence of underage drinking varies by country; however, many countries are experiencing a downward trend [5]. An analysis of the factors related to underage drinking is necessary to plan further countermeasures.

Alcohol advertising is perceived as attractive by some students [6]. Previous studies, mainly in Western countries, have found that underage exposure to alcohol advertisements led the youth who did not consume alcohol to engage in drinking behavior and was positively associated with the consumption of alcoholic beverages [7–14]. A ban on alcohol advertising was reported to reduce recorded alcohol sales, suggesting its protective effect in reducing total alcohol consumption [15]. However, some limitations of prior investigations are (1) it is not well known whether exposure to alcohol advertisements is associated with drinking behavior among adolescents of East Asian populations, including Japan, where the prevalence of underage drinking is lower than that in Western populations (e.g., reported underage drinking rates: 5.6% in Japan in 2017 and 29.2% in the US in 2019) [12, 16–18]; (2) limited studies with large sample sizes (e.g., more than 10,000 participants); and (3) few reports on a graded association between the cumulative number of different advertising media and drinking behavior (dose-response relationship). We hypothesized that current drinkers among Japanese adolescents are exposed to more alcoholic beverage advertising than non-drinkers.

Using data from a nationwide survey conducted in 2021 among junior and senior high school students across Japan, we aimed to clarify the association of exposure to advertising through various media (websites, television, stores, and public transportation) and the cumulative number of different advertising media with drinking behavior. Many countries have declining underage drinking rates, and reports from countries with quite low underage drinking rates, such as present-day Japan, are useful for developing future global strategies and effective advertising control policies to prevent underage drinking [5].

## 2. Methods

### 2.1 Data sources

Drinking behaviors of Japanese junior and senior high school students have been reported in our national survey [1, 16, 17]. The present study used data from the Lifestyle Survey of Adolescents conducted in 2021. Since 1996, our working group for the Lifestyle Survey of Adolescents conducted by the Japanese Ministry of Health, Labour and Welfare, has examined a representative sample of Japanese adolescents using a single stratified single-stage standard cluster sampling method. Following the group’s past methods, we conducted the 2021 survey [1, 16, 17, 19]. The cluster units included junior and senior high schools. This survey method divided Japan into regional blocks and randomly selected schools from each block [19].

### 2.2 Participants

The survey population consisted of seventh to twelfth graders in junior and senior high schools from all over Japan using the National School Directory. All students enrolled in the sample schools comprised the target population. The distribution of school characteristics (e.g., private vs. public) was selected as representative of the survey population [19].

### 2.3 Survey procedure

In 2021, a paper- or web-based survey was conducted using a questionnaire. The questions were the same for both methods. The schools were randomly assigned to participate in either the paper- or web-based survey. Two-thirds and one-third of the schools were assigned to web- or paper-based surveys, respectively. For the paper-based survey, letters were sent to the principals of the selected schools requesting their cooperation along with questionnaires and envelopes for each enrolled student. Classroom teachers in the participating schools explained the survey to their students and assured them of privacy protection. The questionnaire included a statement requesting honest answers and assuring participants that their answers would not be revealed to teachers or parents. Written informed consent was obtained from all participants. In accordance with the Ethical Guidelines for Medical and Health Research Involving Human Subjects, informed consent was obtained from each high school student and the parent or guardian of each junior high school student because this was an anonymous, non-invasive questionnaire survey. The nature of confidentiality and voluntary participation was explained to all students by the teachers, and the completed questionnaires were returned to our working group in sealed envelopes [19]. In the web-based survey, randomly selected schools were asked to cooperate, and students were given a document to take home for their parents regarding participation in the survey. Students who agreed to cooperate participated in the web-based survey using computers and tablets. Anonymous questionnaires were administered to avoid personal identification. This survey was approved by the Institutional Review Boards of Tottori University (No. 20A099, approved on April 30, 2021) and Okayama University (No. K2108-042, approved on August 13, 2021).

### 2.4 Assessment of alcohol advertising media

Using a questionnaire about the exposure to the types of media on alcohol advertising in the past 30 days, we tabulated the exposure to each type of advertising media (websites, television, stores, and public transportation), as well as the cumulative number of different advertising media to which participants were exposed. The question about advertising media was formed by discussing it in the context of Japan based on previous research [11, 20]. The prevalence of exposure to television advertising was high in both non-drinkers and current drinkers (89.4% and 86.5%, respectively); the proportion of non-exposed groups as a reference was low and was, therefore, not suitable for inclusion in the multivariable analysis. Hence, television advertising was excluded from the odds ratio (OR) calculation of the presence of current drinking based on exposure to television advertising.

### 2.5 Alcohol drinking and covariate assessment

For the question about current drinking (“How many days did you consume alcoholic beverages during the past 30 days?”), participants were considered current drinkers if they answered that they consumed alcohol for one or more days. The participants provided data on demographic and school characteristics, including sex, grade, school type (junior or senior high school), and school area. School area was categorized into eastern Japan (Hokkaido-Tohoku and Kanto districts), central Japan (Hokuriku, Koshinetsu, and Tokai districts), and western Japan (Kansai, Chugoku, Shikoku, and Kyushu-Okinawa districts). Participants responded to lifestyle behavior questions, including smoking status and bedtime. For the smoking-related question (“How many days did you smoke during the past 30 days?”), participants were considered current smokers if they answered that they had smoked for one or more days. Bedtime was divided into two categories: before and after 12:00 a.m. Participants were asked if they felt happy at school with the following choices: “Yes, I do,” “Neutral,” and “No, I don’t” [19]. Parental drinking was assessed using the question, “Have you ever felt uncomfortable with your parents drinking alcohol?” Those who answered “Yes” or “No” were categorized as having parents who drank alcohol, and those who answered “parents do not drink alcohol” were categorized as having no parent who drank alcohol. The number of drinking days within the past 30 days and the amount of alcohol consumed per drinking occasion were assessed for current drinkers.

### 2.6 Statistical analysis

Participants’ demographic characteristics were presented as numbers and percentages and were listed by current drinking status and sex; differences in characteristics were evaluated with a chi-square test. We also tabulated the characteristics according to the two survey methods (paper-based or web-based) and examined the difference between them using a chi-square test. We used multivariable logistic regression to calculate the ORs and 95% confidence intervals (CIs) for the presence of current drinking according to the type of media advertising alcohol. The following adjusted models were constructed: Model 1 was adjusted for invariant factors such as sex, grades, and school area (eastern, central, and western Japan), and Model 2 was additionally adjusted for students’ lifestyles (bedtime and having fun at school) and addictive behaviors (smoking status and parents’ alcohol consumption). Covariates considered relevant to underage drinking or advertising exposure were included in the multivariable models; however, school type was not included due to multicollinearity with grades. We repeated the analysis stratified by sex (boys vs. girls) and examined the multiplicative interactions between sex and alcohol advertising for current drinking. To test for systematic errors by survey method (paper-based or web-based), we conducted multivariable analyses stratified by survey method using the same logistic regression models. In addition, we performed a multivariable analysis in which all advertisements (websites, stores, public transportation) were simultaneously adjusted for all advertising media in the multivariable model (other covariates were the same as in Model 2). Furthermore, multivariable analysis by school type (junior or senior high school) using the same logistic regression models was conducted to confirm the differences. Since many current drinkers were smokers (Table 1), the multivariable analysis of only non-smokers was conducted using the same logistic regression models. All probability values were two-tailed, and all CIs were estimated at a 95% level. The level of statistical significance was defined as a two-tailed *p*-value of 0.05 or less. We handled missing data using pairwise deletion. Multicollinearity was assessed using the variance inflation factor (VIF) for all models. All analyses were performed using the STATA statistical software (version 17.0; StataCorp LP, College Station, TX, USA).

**Table 1:** Characteristics of the study participants by current drinking and sex.

	**All**			**Boys**			**Girls**		

	**current drinking**		***p*-value**	**current drinking**		***p*-value**	**current drinking**		***p*-value**
	**(−)**	**(+)**		**(−)**	**(+)**		**(−)**	**(+)**	
	**n = 15,343**	**n = 340**		**n = 7,538**	**n = 177**		**n = 7,805**	**n = 163**	
Type of school, n (% in row)									
Junior high school	8,044 (98.4)	133 (1.6)	<0.001	4,307 (98.1)	82 (1.9)	0.004	3,737 (98.7)	51 (1.3)	<0.001
Senior high school	7,299 (97.2)	207 (2.8)		3,231 (97.1)	95 (2.9)		4,068 (97.3)	112 (2.7)	
Grades, n (% in row)									
7th	2,738 (98.6)	38 (1.4)	<0.001	1,454 (98.1)	28 (1.9)	<0.001	1,284 (99.2)	10 (0.8)	<0.001
8th	2,737 (98.6)	38 (1.4)		1,425 (98.0)	29 (2.0)		1,312 (99.3)	9 (0.7)	
9th	2,569 (97.8)	57 (2.2)		1,428 (98.3)	25 (1.7)		1,141 (97.3)	32 (2.7)	
10th	2,898 (98.5)	43 (1.5)		1,273 (98.8)	15 (1.2)		1,625 (98.3)	28 (1.7)	
11th	2,198 (96.3)	85 (3.7)		976 (96.3)	37 (3.7)		1,222 (96.2)	48 (3.8)	
12th	2,203 (96.5)	79 (3.5)		982 (95.8)	43 (4.2)		1,221 (97.1)	36 (2.9)	
School area, n (% in row)									
Eastern	6,282 (98.1)	122 (1.9)	<0.001	2,979 (98.2)	56 (1.8)	0.033	3,303 (98.0)	66 (2.0)	0.001
Central	4,987 (98.1)	94 (1.9)		2,560 (97.7)	60 (2.3)		2,427 (98.6)	34 (1.4)	
Western	4,074 (97.0)	124 (3.0)		1,999 (97.0)	61 (3.0)		2,075 (97.1)	63 (2.9)	

Bedtime, n (% in row)									
Before 12 AM	9,955 (98.3)	169 (1.7)	<0.001	5,105 (98.2)	94 (1.8)	<0.001	4,850 (98.5)	75 (1.5)	<0.001
After 12 AM	5,355 (97.0)	168 (3.0)		2,411 (96.7)	81 (3.3)		2,944 (97.1)	87 (2.9)	
Having fun at school, n (% in row)									
Yes	10,354 (98.3)	180 (1.7)	<0.001	5,325 (98.2)	99 (1.8)	<0.001	5,029 (98.4)	81 (1.6)	<0.001
Neutral	3,985 (97.6)	100 (2.4)		1,740 (97.4)	47 (2.6)		2,245 (97.7)	53 (2.3)	
No	922 (94.5)	54 (5.5)		424 (94.2)	26 (5.8)		498 (94.7)	28 (5.3)	

Smoking, n (% in column)									
Yes	66 (0.4)	49 (14.6)	<0.001	42 (0.6)	23 (13.1)	<0.001	24 (0.3)	26 (16.1)	<0.001
Parents’ alcohol consumption, n (% in column)									
Yes	13,245 (86.4)	323 (95.0)	<0.001	6,533 (86.7)	167 (94.4)	0.003	6,712 (86.1)	156 (95.7)	<0.001
Frequency of alcohol consumption, n (% in column)									
≤2 days		239 (70.3)			127 (71.8)			112 (68.7)	
≥3 days		101 (29.7)			50 (28.2)			51 (31.3)	
Amount of alcohol, n (% in column)									
Less than a glass		143 (42.2)			86 (48.9)			57 (35.0)	
1 or 2 glasses		121 (35.7)			57 (32.4)			64 (39.3)	
≥3 glasses		75 (22.1)			33 (18.8)			42 (25.8)	

*P*-values were calculated for the chi-square test.

A current drinker was defined as a student who had drunk alcohol for ≥1 day of the 30 days preceding the survey.

Data with the following missing values (partially missing responses) were included: bedtime (36), having fun at school (88), smoking (98), parents’ alcohol consumption (18), frequency of alcohol consumption (23), and amount of alcohol consumption (12).

## 3. Results

Of the 91 junior and 62 senior high schools that were selected, 18 (19.8%) and 17 (27.4%), respectively, participated in this study. Of 95,548 eligible students (45,225 junior and 50,323 senior high school students), 15,832 (8,266 junior and 7,566 senior high school students) responded to the questionnaire survey (valid response rate: 16.6% [18.3% junior and 15.0% senior high school students]). In the present analysis, we excluded 149 students with missing data (categories with missing responses included current drinking, exposure to advertisements, sex, and grades); therefore, data for 15,683 students (7,715 boys and 7,968 girls; 9,396 paper-based survey respondents and 6,287 web-based survey respondents) were analyzed. Data with the following missing values (partially missing responses) were included: bedtime (36), having fun at school (88), smoking (98), parents’ alcohol consumption (18), frequency of alcohol consumption (23), and amount of alcohol consumption (12). Missing values for covariates were duplicated within each participant; the multivariable analyses included data from 15,683 participants for Model 1 and 15,483 participants for Model 2. Table 1 shows the participants’ characteristics according to sex and current drinking status. In our study, the percentage of current drinkers was 2.2% (2.3% of boys, 2.0% of girls). Current drinkers were more prevalent in upper grades, high schools, western Japan, current smokers, those with late bedtimes, and those who did not have fun at school. The prevalence of alcohol advertisements according to current drinking status and sex is shown in Table 2. Current drinkers had a higher prevalence of exposure to alcohol advertisements (i.e., higher exposure to each advertising media and a larger cumulative number of different advertisement media). The prevalence of exposure to at least one alcohol advertisement was 48.8% for non-drinkers and 60.0% for current drinkers. Consistently, for each type of alcoholic beverage advertisement media (websites, stores, public transportation), current drinkers had a higher prevalence of exposure to advertisements than non-drinkers. There were several differences in characteristics between two survey methods (paper-based or web-based) (Supplementary Tables 1 and 2). For example, the web-based survey group included a higher percentage of girls, a higher percentage of students in grades 7 through 10, less exposure to advertising in public transportation (fewer students and a lower percentage of students), and less cumulative number of different advertisement media compared to the paper-based survey group.

**Table 2:** Exposure to alcohol advertisements by current drinking and sex.

	**All**			**Boys**			**Girls**		

	**Current drinking**		***p*-value**	**Current drinking**		***p*-value**	**Current drinking**		***p*-value**
	**(−)**	**(+)**		**(−)**	**(+)**		**(−)**	**(+)**	
	**n = 15,343**	**n = 340**		**n = 7,538**	**n = 177**		**n = 7,805**	**n = 163**	
Advertising media, n (% in column)									
Any media^a^	7,486 (48.8)	204 (60.0)	<0.001	3,872 (51.4)	101 (57.1)	0.134	3,614 (46.3)	103 (63.2)	<0.001
Websites	5,504 (35.9)	151 (44.4)	0.001	3,084 (40.9)	77 (43.5)	0.489	2,420 (31.0)	74 (45.4)	<0.001
Stores	4,199 (27.4)	133 (39.1)	<0.001	1,990 (26.4)	62 (35.0)	0.010	2,209 (28.3)	71 (43.6)	<0.001
Public transportation	1,894 (12.3)	55 (16.2)	0.034	1,074 (14.2)	32 (18.1)	0.151	820 (10.5)	23 (14.1)	0.139
Cumulative number of different advertising media, n (% in column)									
0	7,857 (51.2)	136 (40.0)	<0.001	3,666 (48.6)	76 (42.9)	0.207	4,191 (53.7)	60 (36.8)	<0.001
1	4,372 (28.5)	106 (31.2)		2,207 (29.3)	52 (29.4)		2,165 (27.7)	54 (33.1)	
2	2,117 (13.8)	61 (17.9)		1,054 (14.0)	28 (15.8)		1,063 (13.6)	33 (20.2)	
3	997 (6.5)	37 (10.9)		611 (8.1)	21 (11.9)		386 (4.9)	16 (9.8)	

*P*-values were calculated for the chi-square test.

A current drinker was defined as a student who had drunk alcohol for ≥1 day of the 30 days preceding the survey.

^a^included advertising on websites, stores, or public transportation.

**Fig. 1:**
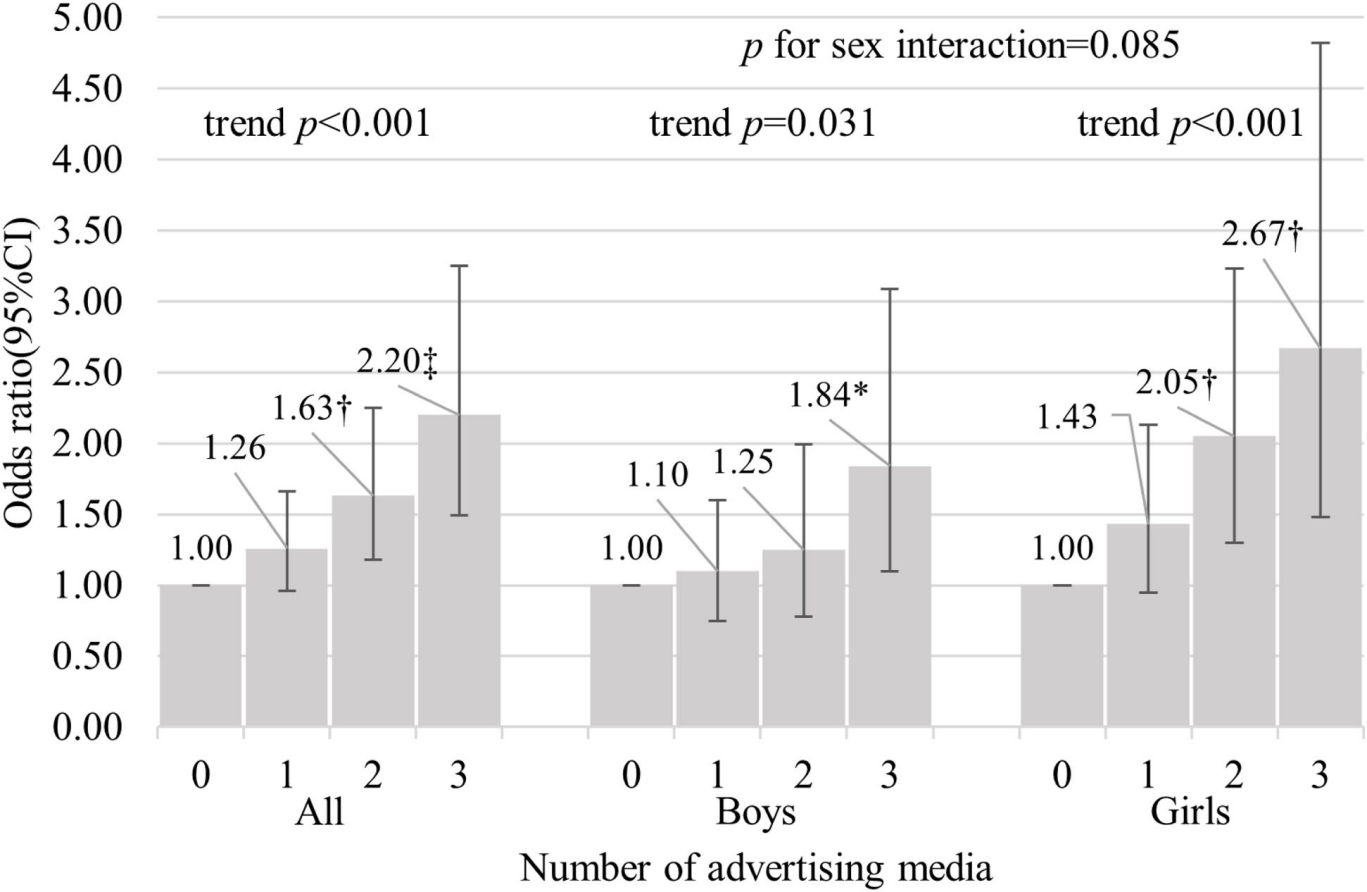
Association between the cumulative number of different alcohol advertising media and current drinking. The number of advertising media is calculated by the cumulative number of different advertising media to which participants were exposed. A current drinker was defined as a student who had drunk alcohol for ≥1 day of the 30 days preceding the survey. The odds ratio of current drinking by the cumulative number of advertising media is shown on the vertical axis. Adjusted for sex, grades, school area, bedtime, having fun at school, smoking status, and parental alcohol consumption. Error bars indicate 95% confidence intervals. CI = confidence interval *p*-value: *<0.05; †<0.01; ‡<0.001.

The association between alcohol advertisement exposure and current drinking is presented in Table 3. After adjustment for sex, grades, and school area (Model 1), students who were exposed to any alcohol advertising media had a higher prevalence of current drinking compared with those who did not (OR 1.67; 95% CI 1.34–2.09). The association persisted after further adjusting for students’ lifestyles (bedtimes and having fun at school) and addictive behavior (smoking status and parents’ alcohol consumption) (OR, 1.48; 95% CI, 1.18–1.87 in Model 2). Students who were exposed to web, in-store, and public transportation advertisements had ORs of 1.44 (95% CI, 1.14–1.81), 1.62 (1.28–2.05), and 1.45 (1.06–1.98) respectively, for the presence of current drinking compared with those who did not, after multivariable adjustment (Model 2). The association of exposure to alcohol advertising media with the presence of current drinking was similar in boys and girls (all *p* values for sex interaction >0.1), except for that of exposure to web advertisements which had a more pronounced association in girls (*p* for sex interaction = 0.046). Exposure to a larger cumulative number of different alcohol advertising media was independently associated with a higher prevalence of current drinking among all students, boys, and girls (*p* values for trend <0.001, 0.031, and <0.001, respectively; *p* for sex interaction = 0.085) (Fig. 1). In the additional analysis conducted to ensure that no obvious systematic errors occurred due to the survey methods, similar results were observed between the two surveys for the association between advertising media and current drinking (all *p* values for interaction >0.5). These results suggest that there are no obvious systematic errors affecting multivariable analysis due to survey methods (Supplementary Table 3 and Supplementary Fig. 1). In a multivariable model that simultaneously adjusted for all advertising media, advertisement in stores was still independently associated with current drinking (Supplementary Table 4). Multivariable analyses of type of school (junior high school or senior high school) and of only non-smokers showed similar results (Supplementary Tables 5 and 6, respectively, and Supplementary Figs. 2 and 3, respectively).

**Table 3:** Association between alcohol advertising media exposure and current drinking.

		**All**	**Boys**	**Girls**	

		**OR (95%CI)**	**OR (95%CI)**	**OR (95%CI)**	***p* for sex interaction**
Any media^a^	Model1	1.67 (1.34–2.09)^‡^	1.36 (1.01–1.85)*	2.05 (1.48–2.84)^‡^	
	Model2	1.48 (1.18–1.87)^†^	1.24 (0.90–1.71)	1.74 (1.23–2.45)^†^	0.105
Websites	Model1	1.53 (1.23–1.91)^‡^	1.19 (0.88–1.61)	1.99 (1.45–2.74)^‡^	
	Model2	1.44 (1.14–1.81)^†^	1.14 (0.83–1.57)	1.80 (1.28–2.52)^†^	0.046
Stores	Model1	1.82 (1.45–2.27)^‡^	1.64 (1.19–2.25)^†^	1.97 (1.44–2.70)^‡^	
	Model2	1.62 (1.28–2.05)^‡^	1.46 (1.04–2.04)*	1.78 (1.28–2.50)^†^	0.289
Public transportation	Model1	1.47 (1.09–1.98)*	1.55 (1.04–2.30)*	1.35 (0.86–2.13)	
	Model2	1.45 (1.06–1.98)*	1.50 (0.99–2.27)	1.39 (0.87–2.22)	0.975

Model 1: adjusted for sex, grades, and school area.

Model 2: additionally adjusted for bedtime, having fun at school, smoking status, and parent’s alcohol consumption.

A current drinker was defined as a student who had drunk alcohol for ≥1 day of the 30 days preceding the survey.

^a^included advertising on websites, stores, or public transportation.

*P* for sex interaction was calculated in Model 2.

OR = odds ratio; CI = confidence interval

*p*-value: *<0.05; †<0.01; ‡<0.001.

## 4. Discussion

In our study based on a nationwide survey of junior and senior high schools across Japan in 2021, we found that only 2.2% of all students currently drank alcohol, and 48.8% of non-drinking and 60.0% of currently drinking adolescents were exposed to at least one or more alcohol advertising media. The prevalence of current drinking among Japanese adolescents from junior and senior high schools in our study has continued to decline in each survey according to the data from a series of nationwide surveys of junior and senior high schools across Japan since 1996 [17]. The underage drinking rate has consistently declined in the present survey in 2021, supporting the reliability of our survey. Exposure to alcohol advertisements on websites, stores, and public transportation was positively associated with current drinking. Statistical analysis of sex differences showed a stronger positive association between web advertisements and current drinking in girls than in boys. Moreover, the odds of current drinking were higher with exposure to a larger cumulative number of different alcohol advertising media; thus, a dose-response relationship between the cumulative number of exposures to different alcohol advertising media and current drinking was revealed. Our study, with a large sample size, is the first to evaluate the relationship between exposure to alcohol advertising through various media (websites, stores, and public transportation) and the cumulative number of different advertising media on drinking behavior among adolescents in Japan, where the prevalence of underage drinking is much lower than that in other countries [16].

Previous studies, mainly in Western countries, have reported adolescents’ exposure to alcohol advertisements and a positive association with drinking behaviors [7, 9, 11–13]. In a cross-sectional analysis of 3,638 Australian adolescents aged 12–17 years, 18% of whom were drinkers and exposure to alcohol advertising via eight types of media (television, internet, sports events, social media, billboard/bus shelter, newspaper, magazine, and radio) was also associated with being a drinker [11]. A two-year prospective study among 3,111 seventh graders in the US found that for 1,206 non-drinkers, several forms of alcohol advertising, including exposure to in-store displays, magazines, and concession stands at sports or music events, predicted underage drinking [7]. Furthermore, a one-year prospective study in Taiwan among 1,795 adolescents aged 13–14 years, 43% of whom experienced alcohol drinking, reported that exposure to advertisements on television predicted an increased likelihood of initiation of alcohol drinking for alcohol-naïve individuals at a one-year follow-up [12]. A possible mechanism in the association between alcohol advertisements and underage drinking is that the advertising attracts adolescents [6]. Our results among Japanese adolescents with low drinking prevalence support the findings of several previous studies, mainly from high-drinking populations, emphasizing that exposure to alcohol advertisements is associated with drinking behavior among adolescents.

The positive association between alcohol advertising, especially on the web, and current drinking was stronger among girls than among boys in the present study. A possible explanation for the gender differences may be that alcohol drinking is a symbol of adulthood for the youth, particularly for boys [21]. For girls, alcohol drinking may have little resonance as a symbol of adulthood; therefore, they were more responsive to imagery in advertisements than boys were [21]. In contrast, alcohol drinking is a symbol of adulthood for boys and may lead to increased participation regardless of variations in advertising levels [21]. In addition, it may be necessary to consider other factors such as affinity for advertising by sex and the ratio of alcohol advertisements to non-alcohol advertisements by advertising medium. Conversely, there have been some reports that boys are more susceptible than girls to alcohol advertising, and no unified view has been reached [22]. Differences in susceptibility to alcoholic beverage advertising between boys and girls could be due to multiple factors and require further investigation. In this study, although we found differences in participant characteristics between the two survey methods, the associations between alcohol advertisements and current drinking in the multivariable analysis were similar for the two survey methods. One possible explanation for the differences, specific to the present study, could be the small number of 11th- and 12th-grade respondents to the web-based survey. The background factors of the difference in characteristics are worthy of further analysis to conduct more effective future research on underage drinking using both paper-based and web-based questionnaire survey (Supplementary Tables 1 and 2).

The prevalence of current drinkers was 2.2% (2.3% of boys and 2.0% of girls) among Japanese adolescents in the present study. A cross-sectional study in 2016 of 3,291 high school students in two prefectures in Japan reported that 9.6% were current drinkers, and our previous cross-sectional study in 2017 of 64,152 junior and senior high school students in a nationwide study reported that 5.6% were current drinkers, indicating a lower drinking rate in the present study in 2021 [17, 23]. According to our several nationwide surveys of junior and senior high school students across Japan, the prevalence of current drinking has been declining since 1996, and the prevalence in the present study is a reasonable result from a chronological perspective [17]. The worldwide prevalence of underage drinking is summarized as follows: 29.2% in the US (2019), 47% in European countries (ESPAD survey, 2019), 23.3% in China (2013–2014), and 19.5% in Taiwan (2006) [18, 24–26]. According to previous studies, the prevalence of alcohol drinking among Japanese adolescents in junior and senior high schools is low compared with that among European and North American adolescents [16]. Although Western countries have higher underage drinking rates than Japan, many countries have declining rates [5]. Reports from countries such as present-day Japan, where underage drinking rates are currently low, are useful to inform future global strategies and establish more effective advertising regulatory policies to prevent underage drinking.

Our study found a positive dose-response relationship between the cumulative number of different alcohol advertising media exposure and current drinking among Japanese adolescents. A dose-response relationship has been reported between past-year exposure to television advertisements and current alcohol consumption in a previous study [9]. Our findings revealed a dose-response relationship with the cumulative number of different advertising media, although a previous study reported this with one media [9]. Our findings suggest that exposure to alcoholic beverage advertising through multiple media sources may promote underage drinking. Considering the positive association between the cumulative number of different advertising media and current drinking in terms of reducing the number of media exposure, it may be effective to prohibit certain media from advertising alcoholic beverages. For example, in accordance with the voluntary standards of the Tobacco Institute of Japan, there are no tobacco advertisements on television or in public transportation. However, Japan has a social background that is more tolerant of alcohol than tobacco [27]. One way of regulating future alcohol advertising would be to refer to tobacco advertising regulations. Based on the results analyzed in the present study, in-store advertising may be the first candidate for regulation (Supplementary Table 4). However, since each advertising medium is associated with current drinking (Table 3), the methods for developing countermeasures to regulate the media must be discussed, and their feasibility and anticipated effects should be measured.

The strengths of our study are its large sample size, its contribution to addressing the scarcity of reports on East Asian populations with low drinking prevalence compared to the West, its representation of Japanese junior and senior high school students (a nationwide survey), being the first to identify a positive relationship between the cumulative number of different alcohol advertising media exposure and current drinking, and exploring sex differences in exposure to advertisements. However, this study has several limitations. First, because this was a cross-sectional study, causal relationships could not be identified. Current drinkers may be more likely to have a lasting impression of alcoholic beverage advertisements than non-drinkers; therefore, current drinkers may have a higher awareness of alcohol advertisements [28]. Consequently, they may have shown a higher rate of alcohol advertisement exposure than non-drinkers. A cohort study is necessary to determine the causal relationships. Second, the valid response rate was low (approximately 15%) possibly because of the social context of the coronavirus disease 2019 (COVID-19) epidemic in Japan and the temporary closure of junior and senior high schools, and so on. The impact of alcohol advertisements on current drinking and the prevalence of underage drinking may have been underestimated because of selection bias. Related to the COVID-19 epidemic, the effect of the fewer opportunities to drink with friends factor on drinking rates could not be examined because of a lack of data as to with whom students share their drinking behavior; hence, further research is needed. Third, due to the low prevalence of current drinking, our study may have been underpowered to detect statistically significant associations between alcohol advertisements and current drinking. Fourth, television advertising was excluded from the present analysis because the prevalence of exposure was high for both drinkers and non-drinkers and was not suitable for analysis. Fifth, we did not collect information about advertisement exposure frequency and exposure time. Sixth, the difference in magnitude for the association with underage drinking between advertising media was not adjusted. Seventh, this is a cross-sectional study, so the possibility of a gap regarding policy implications cannot be ruled out. Eighth, underage drinking is illegal in Japan; thus, social desirability bias is difficult to completely exclude in a survey of adolescents. Ninth, as only students in junior and senior high school in Japan were included in the analysis, our results cannot be generalized to other populations. Finally, the present study may have partly overlooked unmeasurable confounding factors (e.g., daily behavior among adolescents, peer pressure from friends).

## 5. Conclusion

Exposure to alcohol advertisements is positively associated with current drinking among junior and senior high school adolescents in Japan. The association between advertising and current drinking has been confirmed in Japan where underage drinking rates are low. Furthermore, we found that the odds ratios of current drinking were higher with exposure to a larger cumulative number of different alcohol advertising media for both sexes (dose-response relationship). Our results revealed sex differences in the strength of the association between exposure to alcohol advertisements on websites and current drinking. Although further studies are required to determine the relevant factors, and there are possible biases (e.g., social desirability bias), our results reveal an association between alcohol advertising and underage drinking among low underage drinking rate population and highlight the direction for further advertising regulations to prevent underage drinking.

## Abbreviations

OR: odds ratio
CI: confidence interval
VIF: variance inflation factor
ESPAD survey: European School Survey Project on Alcohol and Other Drugs survey
COVID-19: coronavirus disease 2019

## Supplementary information

**Additional file 1:** **Supplementary Table 1.** Characteristics of the study participants by survey methods. **Supplementary Table 2.** Exposure to alcohol advertisements by survey methods. **Supplementary Table 3.** Associations between alcohol advertisement exposure and current drinking by survey methods. **Supplementary Table 4.** Associations between alcohol advertisement exposure and current drinking (simultaneously adjusted for all advertising media). **Supplementary Table 5.** Associations between alcohol advertisement exposure and current drinking (by type of school). **Supplementary Table 6.** Associations between alcohol advertisement exposure and current drinking (non-smokers only). **Supplementary Figure 1.** Associations between the cumulative number of different alcohol advertising media and current drinking (by survey methods). **Supplementary Figure 2.** Associations between the cumulative number of different alcohol advertising media and current drinking (by type of school). **Supplementary Figure 3.** Associations between the cumulative number of different alcohol advertising media and current drinking (non-smokers only).

## Declarations

### Availability of data and material

The datasets analyzed in this survey are not publicly available.
