# Supplementary material for: Association and dose-response relationship between exposure to alcohol advertising media and current drinking: a nationwide cross-sectional study of Japanese adolescents
Source: Environ Health Prev Med. 2023 Sep 28;28:58. doi: 10.1265/ehpm.23-00127 (PMC10569966; doi:10.1265/ehpm.23-00127)
Supplement: Supplementary file 1 — Additional file 1: Supplementary Table 1. Characteristics of the study participants by survey methods. Supplementary Table 2. Exposure to alcohol advertisements by survey methods. Supplementary Table 3. Associations between alcohol advertisement exposure and current drinking by survey methods. Supplementary Table 4. Associations between alcohol advertisement exposure and current drinking (simultaneously adjusted for all advertising media). Supplementary Table 5. Associations between alcohol advertisement exposure and current drinking (by type of school). Supplementary Table 6. Associations between alcohol advertisement exposure and current drinking (non-smokers only). Supplementary Figure 1. Associations between the cumulative number of different alcohol advertising media and current drinking (by survey methods). Supplementary Figure 2. Associations between the cumulative number of different alcohol advertising media and current drinking (by type of school). Supplementary Figure 3. Associations between the cumulative number of different alcohol advertising media and current drinking (non-smokers only). [file ehpm-28-058-s001.pdf]

Supplementary Tables

Supplementary Table 1. Characteristics of the study participants by survey methods

	paper (n=9396)	web (n=6287)	*p*-value
Sex, n (% in column)			
Boys	4822 (51.3)	2893 (46.0)	<0.001
Girls	4574 (48.7)	3394 (54.0)	
Type of school, n (% in column)			
Junior high school	4532 (48.2)	3645 (58.0)	<0.001
Senior high school	4864 (51.8)	2642 (42.0)	
Grades, n (% in column)			
7th	1507 (16.0)	1269 (20.2)	<0.001
8th	1508 (16.0)	1267 (20.2)	
9th	1517 (16.1)	1109 (17.6)	
10th	1626 (17.3)	1315 (20.9)	
11th	1605 (17.1)	678 (10.8)	
12th	1633 (17.4)	649 (10.3)	
School area, n (% in column)			
Eastern	5123 (54.5)	1281 (20.4)	<0.001
Central	1579 (16.8)	3502 (55.7)	
Western	2694 (28.7)	1504 (23.9)	

Bedtime, n (% in column)

Before 12 AM	5932 (63.4)	4192 (66.7)	<0.001
After 12 AM	3428 (36.6)	2095 (33.3)	
Having fun at school, n (% in column)			
Yes	6344 (68.2)	4190 (66.6)	0.130
Neutral	2398 (25.8)	1687 (26.8)	
No	566 (6.1)	410 (6.5)	
Current drinking, n (% in column)			
Yes	231 (2.5)	109 (1.7)	0.002
Smoking, n (% in column)			
Yes	80 (0.9)	35 (0.6)	0.030
Parents' alcohol consumption, n (% in column)			
Yes	8124 (86.6)	5444 (86.6)	0.947
Frequency of alcohol consumption, n (% in column)			
≤2 days	9296 (99.2)	6263 (99.6)	0.001
≥3 days	77 (0.8)	24 (0.4)	
Amount of alcohol, n (% in column)			
Less than a glass	9076 (96.7)	6158 (97.9)	<0.001
1 or 2 glasses	223 (2.4)	102 (1.6)	
≥3 glasses	85 (0.9)	27 (0.4)	

P-values were calculated for the chi-square test.

A current drinker was defined as a student who had drunk alcohol for ≥1 day of the 30 days preceding the survey.

Data with the following missing values (partially missing responses) were included: bedtime (36), having fun at school (88), smoking (98), parents'

alcohol consumption (18), frequency of alcohol consumption (23), and amount of alcohol consumption (12).

Supplementary Table 2. Exposure to alcohol advertisements by survey methods

	paper (n=9396)	web (n=6287)	*p*-value
Advertising media, n (%) in column			
Any media[a]	4697 (50.0)	2993 (47.6)	0.003
Website	3363 (35.8)	2292 (36.5)	0.396
Stores	2547 (27.1)	1785 (28.4)	0.078
Public transportation	1333 (14.2)	616 (9.8)	<0.001
Cumulative number of different advertising media, n (% in column)			
0	4699 (50.0)	3294 (52.4)	0.001
1	2787 (29.7)	1691 (26.9)	
2	1274 (13.6)	904 (14.4)	
3	636 (6.8)	398 (6.3)	

P-values were calculated for the chi-square test.

[a]included advertising on websites, stores, or public transportation.

Supplementary Table 3. Associations between alcohol advertisement exposure and current drinking by survey methods

		paper (n=9396)	web (n=6287)	
		OR (95%CI)	OR (95%CI)	*p* for interaction
Any media[a]	Model1	1.69 (1.29-2.22)‡	1.60 (1.08-2.35)*	
	Model2	1.48 (1.11-1.97)†	1.43 (0.96-2.14)	0.908
Websites	Model1	1.54 (1.18-2.01)†	1.50 (1.02-2.21)*	
	Model2	1.45 (1.09-1.93)*	1.39 (0.94-2.07)	0.950
Stores	Model1	1.92 (1.47-2.52)‡	1.59 (1.07-2.37)*	
	Model2	1.69 (1.27-2.26)‡	1.47 (0.98-2.20)	0.503
Public transportation	Model1	1.40 (0.97-2.03)	1.50 (0.88-2.56)	
	Model2	1.37 (0.93-2.02)	1.45 (0.84-2.50)	0.712

Model 1: adjusted for sex, grades, and school area.

Model 2: additionally adjusted for bedtime, having fun at school, smoking status, and parents' alcohol consumption.

A current drinker was defined as a student who had drunk alcohol for ≥1 day of the 30 days preceding the survey.

[a]included advertising on websites, stores, or public transportation.

P for interaction was calculated with Model 2.

OR = odds ratio; CI = confidence interval

p-value: * < 0.05; † < 0.01; ‡ < 0.001.

Supplementary Table 4. Associations between alcohol advertisement exposure and current drinking (simultaneously adjusted for all advertising media)

	All	Boys	Girls	
	OR (95%CI)	OR (95%CI)	OR (95%CI)	*p* for sex interaction
Websites	1.25 (0.98-1.61)	0.99 (0.70-1.40)	1.57 (1.10-2.25)*	0.040
Stores	1.46 (1.12-1.90)†	1.35 (0.92-1.99)	1.56 (1.08-2.24)*	0.236
Public transportation	1.12 (0.80-1.57)	1.29 (0.81-2.04)	1.00 (0.60-1.64)	0.860

All advertising media were included simultaneously in the multivariable model.

Adjusted for each advertising media, sex, grades, school area, bedtime, having fun at school, smoking status, and parents' alcohol consumption.

A current drinker was defined as a student who had drunk alcohol for ≥1 day of the 30 days preceding the survey.

OR = odds ratio; CI = confidence interval

p-value: * < 0.05; † < 0.01; ‡ < 0.001.

Supplementary Table 5. Associations between alcohol advertisement exposure and current drinking (by type of school)

		junior high school	senior high school	
		OR(95%CI)	OR(95%CI)	*p* for interaction
Any media[a]	Model1	1.75(1.22-2.52)[†]	1.63(1.23-2.16)[†]	
	Model2	1.53(1.05-2.23)[*]	1.43(1.06-1.93)[*]	0.887
Website or video site	Model1	1.64(1.16-2.31)[†]	1.47(1.10-1.96)[†]	
	Model2	1.50(1.04-2.15)[*]	1.39(1.02-1.79)*	0.660
In-store Poster	Model1	1.83(1.29-2.59)[†]	1.79(1.34-2.40)[‡]	
	Model2	1.68(1.17-2.41)[†]	1.54(1.12-2.11)[†]	0.838
public transportation	Model1	1.95(1.26-3.01)[†]	1.16(0.76-1.76)	
	Model2	1.77(1.13-2.78)[*]	1.22(0.79-1.89)	0.232

Model 1: adjusted for sex, grades, and school area.

Model 2: additionally adjusted for bedtime, having fun at school, smoking status, and parents' alcohol consumption.

A current drinker was defined as a student who had drunk alcohol for ≥1 day of the 30 days preceding the survey.

[a]included advertising on websites, stores, or public transportation.

P for interaction was calculated with Model 2.

OR = odds ratio; CI = confidence interval

p-value: ＊< 0.05; † < 0.01; ‡ < 0.001.

Supplementary Table 6. Associations between alcohol advertisement exposure and current drinking (non-smokers only)

		non-smoker (n=15470)
		OR (95%CI)
Any media[a]	Model1	1.48 (1.17-1.88)†
	Model2	1.43 (1.12-1.82)†
Web	Model1	1.46 (1.15-1.85)†
	Model2	1.40 (1.10-1.78)†
In-store	Model1	1.66 (1.30-2.11)‡
	Model2	1.62 (1.27-2.07)‡
Public transportation	Model1	1.45 (1.04-2.00)†
	Model2	1.38 (0.99-1.91)

Model 1: adjusted for sex, grades, and school area.

Model 2: additionally adjusted for bedtime, having fun at school, smoking status, and parents' alcohol consumption.

A current drinker was defined as a student who had drunk alcohol for ≥1 day of the 30 days preceding the survey.

[a]included advertising on websites, stores, or public transportation.

OR = odds ratio; CI = confidence interval

p-value: ＊< 0.05; † < 0.01; ‡ < 0.001.

Supplementary Figures

Supplementary Figure 1. Associations between the cumulative number of different alcohol advertising media and current drinking (by survey methods)

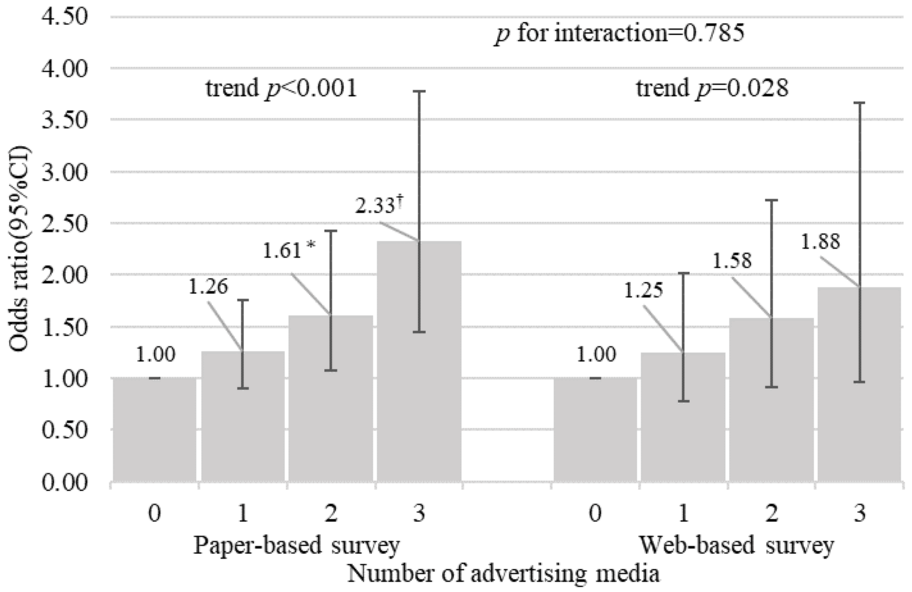

The number of advertising media is calculated by the cumulative number of different advertising media to which participants were exposed.

A current drinker was defined as a student who had drunk alcohol for ≥1 day of the 30 days preceding the survey.

The odds ratio of current drinking by the cumulative number of advertising media is shown on the vertical axis.

Adjusted for sex, grades, school area, bedtime, having fun at school, smoking status, and parental alcohol consumption.

Error bars indicate 95% confidence intervals.

CI = confidence interval

p-value: *< 0.05; †< 0.01; ‡< 0.001.

Supplementary Figure 2. Associations between the cumulative number of different alcohol advertising media and current drinking (by type of school)

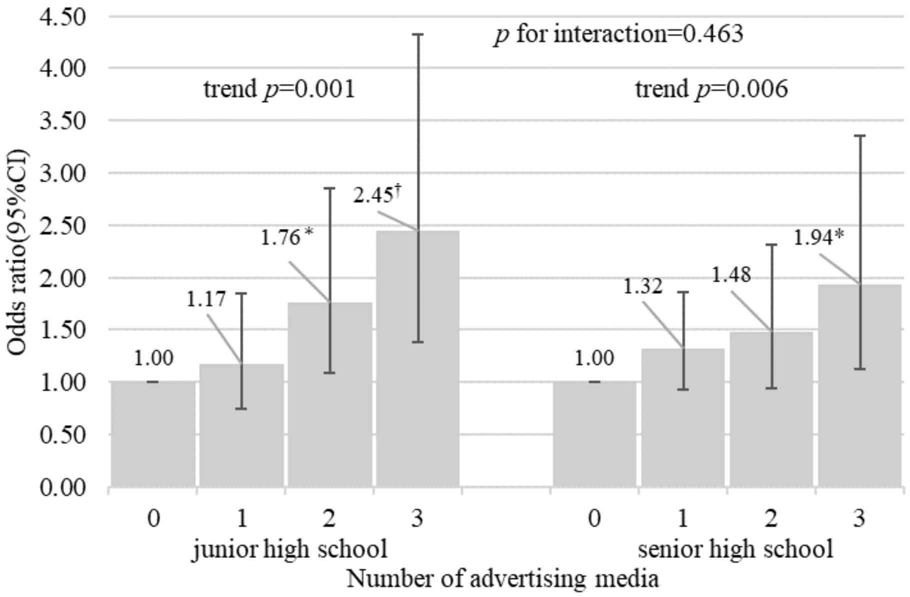

The number of advertising media is calculated by the cumulative number of different advertising media to which participants were exposed.

A current drinker was defined as a student who had drunk alcohol for ≥1 day of the 30 days preceding the survey.

The odds ratio of current drinking by the cumulative number of advertising media is shown in the vertical axis.

Adjusted for sex, grades, school area, bedtime, having fun at school, smoking status, and parental alcohol consumption.

Error bars indicate 95% confidence intervals.

CI = confidence interval

p-value: *< 0.05; †< 0.01; ‡< 0.001.

Supplementary Figure 3. Associations between the cumulative number of different alcohol advertising media and current drinking (non-smokers only)

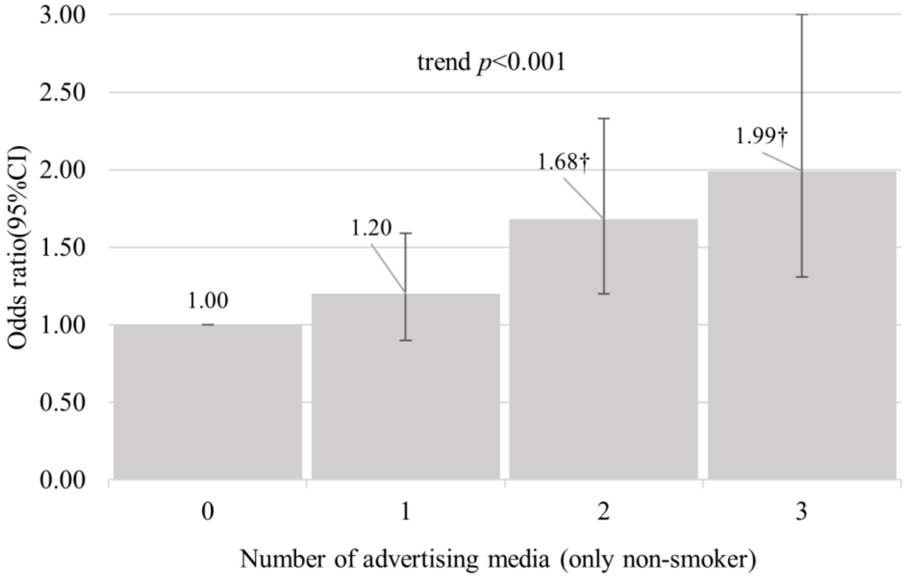

Number of advertising media is calculated by the cumulative number of different advertising media to which participants were exposed.

A current drinker was defined as a student who had drunk alcohol for ≥1 day of the 30 days preceding the survey.

The odds ratio of current drinking by the cumulative number of advertising media is shown in the vertical axis.

Adjusted for sex, grades, school area, bedtime, having fun at school, smoking status, and parental alcohol consumption.

Error bars indicate 95% confidence intervals.

CI = confidence interval

p-value: *< 0.05; †< 0.01; ‡< 0.001.
